# Total laparoscopic limited anatomical resection for centrally located hepatocellular carcinoma in cirrhotic liver

**DOI:** 10.1007/s00464-012-2624-6

**Published:** 2012-12-12

**Authors:** Cheng-Maw Ho, Go Wakabayashi, Hiroyuki Nitta, Masahiro Takahashi, Takeshi Takahara, Naoko Ito, Yasushi Hasegawa

**Affiliations:** 1 Department of Surgery, Iwate Medical University School of Medicine, 19-1 Uchimaru, Morioka, Iwate 020-8505 Japan; 2 Department of Surgery, National Taiwan University Hospital, Taipei, Taiwan

**Keywords:** Limited anatomical resection, Laparoscopy, Hepatocellular carcinoma, Cirrhosis

## Abstract

**Background:**

Limited anatomical liver resection for hepatocellular carcinoma (HCC) is complicated in cirrhotic patients with centrally located HCC and limited liver reserve. We present a case of total laparoscopic left medial and right ventroanterior sectionectomy performed using the intrahepatic Glissonian approach in a cirrhotic liver for curative resection of HCC.

**Methods:**

The patient was a 69-year-old man with a 6.5-cm-diameter HCC located at segments 4, 5, and 8 and which was compressing the middle hepatic vein (MHV). Child–Pugh class A liver cirrhosis was noted, and the 15-min retention rate for indocyanine green was 14 %. Preoperative surgical planning suggested the feasibility of limited anatomical subsegmental resection. The patient was placed in the supine position and 5 trocars were used for the procedure. The operation began with cholecystectomy, division of liver ligaments, and exposure of the right hepatic vein root and the umbilical Glissonian pedicles to the left medial segment. Parenchymal transection was performed using a laparoscopic harmonic scalpel and Cavitron Ultrasonic Surgical Aspirator until the MHV was reached. After exposing the ventral branches of the right anterior Glissonian pedicle and dividing them, resection was continued along the demarcation line. Fissure veins draining to the MHV root were identified and divided. The MHV root was closed using an automatic stapler.

**Results:**

The operation time was 565 min and estimated blood loss was 665 ml; blood transfusion was not required. Pathological examination confirmed a moderately differentiated HCC with all resected margins free of malignancy. Postoperative recovery was uneventful and the patient was discharged on the postoperative day 7. There was no tumor recurrence 18 months after the operation.

**Conclusions:**

Total laparoscopic left medial and right ventroanterior sectionectomy via the intrahepatic Glissonian approach is feasible for HCC in a cirrhotic liver with limited liver reserve. Preoperative planning is essential in order to compute successful hepatic function. Standardization of surgical techniques may aid in safely performing this procedure.

**Electronic supplementary material:**

The online version of this article (doi:10.1007/s00464-012-2624-6) contains supplementary material, which is available to authorized users.

Laparoscopic liver resection, which was first reported in 1992 [1], is gaining increasing acceptance and is often applied as the surgical treatment of hepatocellular carcinoma (HCC). A previous study conducted on a selected group of patients showed that the 5-year survival rate of patients who underwent laparoscopic HCC resection was comparable to that of patients who underwent open hepatic resection [2]. Compared to nonanatomic resection, anatomic resection for a single HCC is known to yield a more favorable outcome [3], which holds true also for anatomic subsegmentectomy [4]. However, cirrhosis is present in over 80 % of patients with HCC [5]. Maintaining adequate liver function during resection is extremely important for patient survival [6]. In HCC patients, particularly those with a large HCC, it is difficult to safely perform laparoscopic anatomic resection with an adequate resection margin [7]. Therefore, laparoscopic liver resection for HCC is limited to highly selected cases [8].

Laparoscopic left medial and right anterior sectionectomy (central hepatectomy) has rarely been performed, with only nine cases reported to date [2, 9, 10]. Among these cases, two involved purely laparoscopic resection: in one case, an otherwise healthy 43-year-old man underwent liver resection for colorectal metastasis [9], and in the other case, the patient underwent resection for a small HCC [10]. To our knowledge, there is no report on totally laparoscopic left medial and right ventroanterior sectionectomy (segment 4, subsegments 5 and 8). Here, we present the case of a patient with a large HCC who was successfully treated using limited anatomic resection of the left medial and right ventroanterior liver segments via an intrahepatic Glissonian approach.

## Patient and methods

**Fig. 1:**
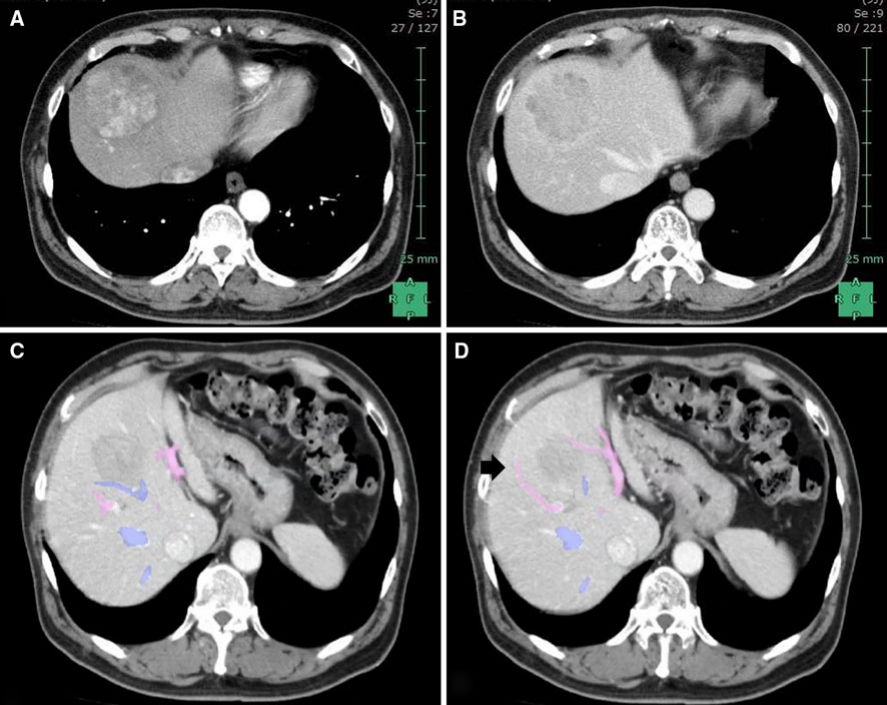
Enhanced abdominal computed tomographic image showing **A** early arterial phase of HCC centrally located in the liver and **B**, **C** compression of the MHV. **D** Ventral branch of the right anterior portal trunk is pointed to by the *arrow*. *Pink* portal vein, *blue* hepatic vein (Color figure online)

**Fig. 2:**
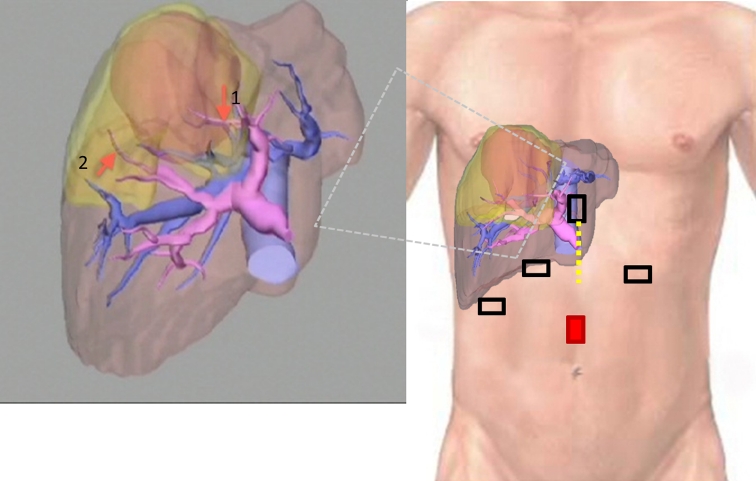
Preoperative surgical planning using 3D liver volumetry and port setting. Calculated volumes of liver (*coffee color*) and tumor (*orange*) were 1,067 and 144 ml, respectively. The tumor compressed the MHV and occupied the parenchyma supplied by the S4 Glissonian (*arrow 1*) and the ventral branch (*arrow 2*) of the right anterior Glissonian pedicles. The proposed resection area, estimated at 260 ml (24 % total liver volume), is shown in *yellow*, which covers the tumor completely. Five ports were required for totally laparoscopic left medial and right ventroanterior segmentectomy. The camera port is in red. All ports, except the left subcostal port, were 12 mm in diameter. The portal vein is shown in *purple*; the hepatic veins and IVC are in *blue*. Incision for minilaparotomy is shown as the *yellow dotted line* (Color figure online)

A 68-year-old man with a history of diabetes mellitus and hypertension, but without hepatitis B or C virus infection, was sonographically confirmed to have a large hepatic tumor during follow-up. He had undergone exploratory laparotomy for colon diverticulitis 7 years prior. Abdominal computed tomography revealed a 6.5-cm liver mass, which showed typical HCC features of early arterial enhancement and portal venous washout, located at hepatic segments 4, 5, and 8 and compressing the middle hepatic vein (MHV, Fig. 1A–C). The tumor was supplied by the ventral branches of the right anterior Glissonian pedicle (Fig. 1D). Preoperative evaluation of the liver reserve revealed Child–Pugh class A liver cirrhosis and the 15-min retention rate of indocyanine green (ICG_15_) was 14 %. The serum levels of tumor markers for alpha-fetoprotein and protein induced by vitamin K absence or antagonist-II were 2.6 ng/ml and 132 mAU/ml, respectively. Preoperative surgical planning using the three-dimensional volume analyzer Synapse Vincent™ (FUJIFILM Co., Japan) suggested the feasibility of limited anatomic subsegmental resection (Fig. 2) instead of central bisegmentectomy (which consisted of 493-ml volume, 46.2 % total liver volume) according to Makuuchi’s criteria [11].

**Fig. 3:**
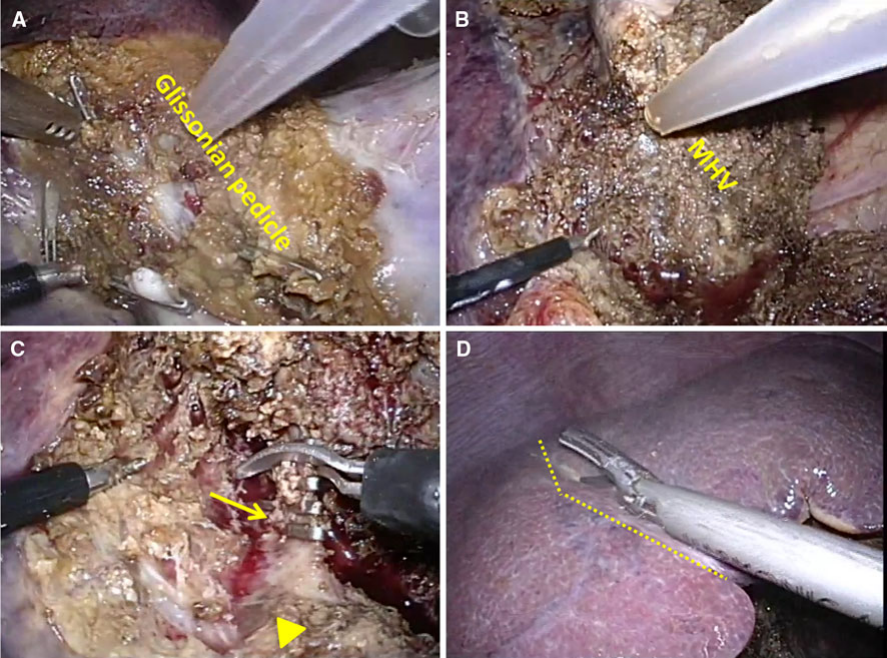
The surgical strategy adopted during total laparoscopic medial and ventroanterior sectionectomy with intraoperative views of the major steps. **A** Left umbilical Glissonian pedicle, which was divided using a vascular endoscopic stapler. **B** Parenchymal transection of the left medial segment toward the right liver lobe. The MHV is shown. **C** The right ventral branch of the anterior Glissonian pedicle (*arrow*) was divided using vascular endoscopic clips, leaving the dorsal branch intact (*arrowhead*). **D** The demarcation line appeared after dividing ventral branches of the right anterior Glissonian pedicle, which was shifted toward the right side from the Cantlie line (*broken line*)

**Fig. 4:**
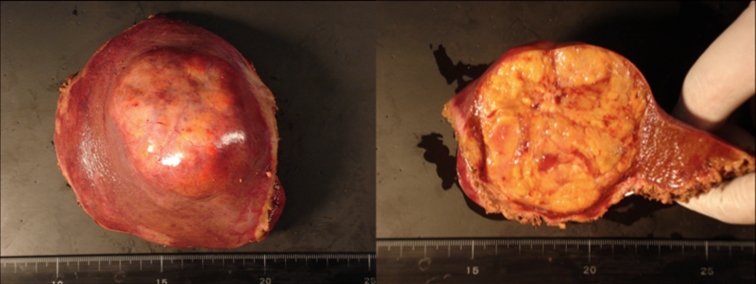
The resected specimen (weight 320 g), showing a 6.5-cm-diameter HCC with negative resection margins

We performed total laparoscopic resection of the left medial (segment 4) and right ventroanterior (subsegments 5 and 8) liver segments. The patient was placed in the supine position with the surgeon standing on the right side of the patient. Five trocars were used as shown in Fig. 2. The operation began with adhesiolysis and cholecystectomy. The right triangular and falciform ligaments were divided from the medial to the lateral side in order to avoid any damage to the inferior vena cava (IVC) and for safely locating the root of the right hepatic vein (RHV). Intraoperative sonographic examination was used to confirm the exact tumor location and its relationship to the major blood vessels. Then, parenchymal transection was performed along the medial side of the falciform ligament, thereby exposing the umbilical Glissonian pedicles, which included the arterial, portal, and bile duct branches of segment 4 and which were clipped and divided (Fig. 3A). The Cantlie line demarcation of the liver lobes was identified and the parenchymal resection was continued toward the right liver lobe using specialized surgical instruments (harmonic scalpel for superficial parenchyma and laparoscopic Cavitron Ultrasonic Surgical Aspirator [CUSA; Valleylab, Boulder, CO] for deep parenchymal tissue), until the level of the MHV (Fig. 3B). The ventral branches of the right anterior Glissonian pedicle were identified, clipped, and divided (Fig. 3C). Furthermore, the new demarcation line, which leaves segments 6 and 7 and dorsal subsegments 5 and 8 intact, appeared (Fig. 3D). Right ventroanterior sectionectomy was then performed. Meticulous transection was necessary to avoid massive bleeding from the transected surface. As the transection proceeded toward the root of the MHV, we encountered the drainage vein from segment 8 (fissure vein) and had to divide the vein. The root of the MHV was closed using an automatic stapler and intraoperative sonography was performed to confirm the integrity of the RHV root. The tumor specimen was removed via minilaparotomy (Fig. 4). Because slight bile spillage was observed from the exposed right anterior Glissonian pedicle, fibrin glue and sealant patch (TachoSil^®^, Nycomed, Linz, Austria) were applied topically to the surface of the bile duct for cover reinforcement after inserting a bile drainage tube (5-French ureteric catheter), which was placed into the remnant cystic duct by minilaparotomy as shown in Fig. 2.

## Results

The operation time was 565 min, the estimated blood loss was 665 ml, and there was no need for blood transfusion. The bile drainage tube was removed on the postoperative day 4. Postoperative recovery was uneventful and the patient was discharged on the postoperative day 7. Pathological examination of the tumor specimen confirmed a stage II moderately differentiated HCC with negative resection margins. The patient showed no tumor recurrence during the follow-up 18 months after surgery.

## Discussion

Laparoscopic surgery is popular because it offers benefits such as a small operative wound and a fast recovery. Anatomic liver resections such as right or left hepatectomy via laparoscopic procedures have been previously reported [2]. However, to our knowledge, only two case reports of pure laparoscopic central bisegmentectomy have been performed: one for a patient with liver metastasis [9] and the other for a patient with small HCC [10]. Here, we demonstrated the feasibility of performing total laparoscopic left medial and right ventroanterior sectionectomy for a 6.5-cm-diameter HCC in a Child–Pugh class A cirrhotic patient. The indications for performing laparoscopic liver resection could be extended to centrally located HCC in a mildly cirrhotic background, which is similar to that for open surgery, so that both anatomic liver resection and maintenance of liver reserve could be adequately achieved. Actuarial preoperative planning is essential to compute successful hepatic function. Further advancements in this technique may enable an increasing number of HCC patients to benefit from this procedure.

Left medial and right anterior sectionectomy is a safe and effective procedure for the treatment of centrally located HCC [12]. Although this procedure is technically demanding, a greater amount of nontumor liver parenchyma is preserved, which is important for the survival of patients with liver cirrhosis. While performing both open surgery and left medial and right anterior sectionectomy, the major difficulty is in safely dissecting the two parenchymal transection planes and in simultaneously preserving the vascular structures that drain or supply the remnant liver [10]. The same principles, though even much more challenging, apply for left medial and right ventroanterior sectionectomy. Detailed anatomical knowledge of the branches of the Glissonian pedicle to the right paramedian segments (segment 5 and 8) is key to performing limited anatomical subsegmental resection [13]. Parenchymal transection in open subsegmentectomy could be guided by ultrasonically guided portal vein puncture and injection of dye which marks the demarcation line [14]. In laparoscopic subsegmentectomy, which is difficult to do, the substitute maneuver is the identification and division of the intrahepatic Glissonian pedicles. Careful dissection and proper surgical control of all the vascular structures are critical for safe and successful resection, particularly in the hilum and the deep portion around the hepatic veins and IVC [10]. There were four critical steps for successful completion of this procedure: surgical control of the intrahepatic Glissonian pedicles (left medial and the ventral branch of the right anterior pedicle), identification of the MHV as parenchymal transection proceeded, division of fissure veins draining to the MHV, and division of the MHV root. Standardization of the surgical techniques is important for facilitating this procedure. Laparoscopic CUSA is very helpful for the detection and isolation of deep-seated vascular structures. Experience in performing a one-plane laparoscopic transection may help surgeons safely perform this two-plane laparoscopic transection. We eagerly anticipate further development of this procedure for increased chances of treatment for HCC patients.

## Conclusions

Total laparoscopic left medial and right anterior sectionectomy is feasible for cirrhotic patients with HCC when residual liver volume is limited. Preoperative planning is essential in order to compute successful hepatic function. The intrahepatic Glissonian approach is useful for this complex procedure. Standardization of surgical techniques may help laparoscopic surgeons safely perform this challenging procedure.

## Electronic supplementary material

Below is the link to the electronic supplementary material.

Supplementary material 1 (MPG 28,3726 kb)
